# Ligand-induced conformational changes in a thermophilic ribose-binding protein

**DOI:** 10.1186/1472-6807-8-50

**Published:** 2008-11-19

**Authors:** Matthew J Cuneo, Lorena S Beese, Homme W Hellinga

**Affiliations:** 1 The Department of Biochemistry, Duke University Medical Center, Durham, North Carolina, 27710, USA

## Abstract

**Background:**

Members of the periplasmic binding protein (PBP) superfamily are involved in transport and signaling processes in both prokaryotes and eukaryotes. Biological responses are typically mediated by ligand-induced conformational changes in which the binding event is coupled to a hinge-bending motion that brings together two domains in a closed form. In all PBP-mediated biological processes, downstream partners recognize the closed form of the protein. This motion has also been exploited in protein engineering experiments to construct biosensors that transduce ligand binding to a variety of physical signals. Understanding the mechanistic details of PBP conformational changes, both global (hinge bending, twisting, shear movements) and local (rotamer changes, backbone motion), therefore is not only important for understanding their biological function but also for protein engineering experiments.

**Results:**

Here we present biochemical characterization and crystal structure determination of the periplasmic ribose-binding protein (RBP) from the hyperthermophile *Thermotoga maritima *in its ribose-bound and unliganded state. The *T. maritima *RBP (tmRBP) has 39% sequence identity and is considerably more resistant to thermal denaturation (^*app*^*T*_*m *_value is 108°C) than the mesophilic *Escherichia coli *homolog (ecRBP) (^*app*^*T*_*m *_value is 56°C). Polar ligand interactions and ligand-induced global conformational changes are conserved among ecRBP and tmRBP; however local structural rearrangements involving side-chain motions in the ligand-binding site are not conserved.

**Conclusion:**

Although the large-scale ligand-induced changes are mediated through similar regions, and are produced by similar backbone movements in tmRBP and ecRBP, the small-scale ligand-induced structural rearrangements differentiate the mesophile and thermophile. This suggests there are mechanistic differences in the manner by which these two proteins bind their ligands and are an example of how two structurally similar proteins utilize different mechanisms to form a ligand-bound state.

## Background

Bacterial periplasmic binding proteins (PBP) are receptors for extracellular solutes in metabolite uptake [1], chemotaxis [2], and intercellular communication [3] processes. The PBPs collectively constitute a structural protein superfamily characterized by two pseudo-symmetric domains that are linked by a hinge formed by two or three β-strands connecting the domains; a ligand-binding site is situated at the interface between the two domains [4]. Each domain adopts a three-layered α/β/α sandwich fold and is classified into one of three structural sub-categories (group I/ribose-binding protein fold, group II/maltose-binding protein fold, and group III/Vitamin B12-binding protein fold) [5] according to β-strand topology.

Ligand-free PBPs adopt an open conformation in which the inter-domain interface is exposed to solvent. Solute binding induces a conformational change to form a closed state in which the ligand is bound at the domain interface and buried by the surrounding protein [6-8]. This closed form typically binds to other molecular components to trigger downstream cellular processes such as chemotaxis [9], quorum sensing [3], and transmembrane ligand transport [10]. Eukaryotic receptors that contain the PBP fold as part of multi-domain proteins are also regulated by ligand-induced conformational coupling mechanisms [11].

A collection of PBP structures determined in both apo and ligand-bound states (Table 1 and references therein) has provided a wealth of information on the ligand-induced domain motions of PBPs. Analysis of the ligand-induced conformational changes in PBPs has to differentiate between different types of motions: large-scale (interdomain) movements, loop movements, relative intradomain movements of secondary structure elements, and amino acid side-chain reorganization. Large-scale changes in PBPs can be described as a rigid body motion of the two domains, characterized by bending/twisting motions around two axes [12]. The magnitude of this hinge-bending motion ranges from 62° in a mutant *E. coli *ribose-binding protein [8] to as little as 14° in the leucine-binding protein [13]. PBPs such as the *E. coli *ribose-binding protein (RBP) [8] and allose-binding protein [7] have been shown to adopt a series of intermediate values in their apo state suggesting that the observed states represent snapshots of a continuum between two extremes: the defined closed form, and a less precisely defined fully open conformation.

**Table 1:** PBPs that have structures of both ligand-bound and ligand-free forms.

		**PDB Entry**			
	**Protein**	**Apo**	**Complex**	**Reference**	**Hinge Bending**[25]

**Group I**	*Escherichia coli *leucine-binding protein	1USG	1USI	Magnusson 2004[13]	14°
	*Escherichia coli *lactose repressor core	1TLF	1LBI	Friedman 1995[42]; Lewis 1996[43]	15°
	*Salmonella typhimurium *autoinducer precursor-binding protein	1TM2	1TJY	Miller 2004[28]	21°
	*Neisseria gonorrhoeae *ferric-binding protein	1R1N	1D9Y	Zhu 2003[44]; McCree Unpublished	24°
	*Thermotoga maritima *ribose-binding protein	2FN9	2FN8	This Work	28° *
	*Escherichia coli *allose-binding protein	1GUD	1RPJ	Chaudhuri 1999[45]; Magnusson 2002[7]	31° *
	*Escherichia coli *glucose-binding protein	2FW0	2FVY	Borrok 2007[46]	31°
	*Thermotoga maritima *glucose/xylose-binding protein	3C6Q	2H3H	Cuneo unpublished	38°
	*Escherichia coli *ribose-binding protein	1URP	2DRI	Bjorkman 1998[8]; Bjorkman 1994[24]	43° *

**Group II**	*Rhodobacter sphaeroides *α-keto acid-binding protein	2HZK	2HZL	Gonin 2007[47]	15°
	*Escherichia coli *nickel-binding protein	1UIU	1UIV	Heddle 2003[48]	17°
	*Homo sapiens *glutamate receptor	1SYH	1N0T	Frandsen 2005[49]; Hogner 2003[50]	18°
	*Haemophilus influenzae *ferric-binding protein	1D9V	1MRP	Bruns 2001[51]; Bruns 1997[52]	20°
	*Haemophilus influenzae *sialic acid-binding protein	2CEY	2CEX	Muller 2006[53]	25°
	*Salmonella typhimurium *oligopeptide-binding protein	1RKM	1RKM	Sleigh 1997[54]	26°
	*Escherichia coli *phosphate-binding protein	1OIB	1QUK	Yao 1996[55]	26°
	*Mannheimia haemolytica *ferric iron-Binding Protein	1SI1	1SI0	Shouldice 2004[56]	27°
	*Vibrio harveyi *autoinducer-binding protein	1ZHH	1JX6	Neiditch 2005[57]; Chen 2002[58]	27°
	*Escherichia coli *maltose-binding protein	1OMP	1ANF	Sharff 1992[14]; Quiocho 1997[59]	36°
	*Sphingomonas sp*. alginate-binding protein	1Y3Q	1Y3N	Momma 2005[60]	39°
	*Yersinia enterocolitica *hexuronate-binding protein	2UVG	2UVH	Abbot 2007[61]	44°
	*Salmonella typhimurium *lysine/arginine/ornithine-binding protein	2LAO	1LST	Oh 1993[62]	52°
	*Escherichia coli *dipeptide-binding protein	1DPE	1DPP	Nickitenko 1995[63]; Dunten 1995[64]	54°
	*Thermotoga maritima *maltotriose-binding protein	2GHB	2GHA	Cuneo unpublished	54°
	*Escherichia coli *glutamine-binding protein	1GGG	1WDN	Hsiao 1996[65]; Sun 1998[66]	56°

PBPs which have been found to adopt multiple open forms (>5° difference) are indicated with an asterisk.

In *E. coli *RBP (ecRBP) small-scale backbone movements are restricted to the hinge region, whereas the secondary structure elements in the two domains and the amino acids in the binding pocket adopt essentially the same conformations in both the apo and ribose-bound forms [8]. However, in *E. coli *leucine-binding protein, not only the hinge region, but also loops and amino acid side-chains in the binding pocket show ligand-induced changes [13], many of which are restricted to one domain. This difference in conformational changes between the domains has been postulated to imply ordered interactions between the protein and ligand [8,13,14].

The ligand-induced conformational changes have not been described previously in a thermophilic PBP. We have characterized the stability, determined the ligand-binding properties, and solved the X-ray crystal structures of the apo and ligand-bound forms of a thermophilic periplasmic ribose-binding protein from the hyperthermophile *Thermotoga maritima *(tmRBP), the mesophilic homolog, ecRBP, of which has been studied in detail [8,15,16]. The ecRBP and tmRBP proteins share 39% amino acid sequence identity, but differ by 52°C in apparent thermal stability. We find that the interdomain motions, although not of the same magnitude, exhibit similar movements. The amino acids in the tmRBP sugar-binding pocket undergo ligand-induced conformational changes, whereas their conformations in apo ecRBP are essentially pre-formed for ligand binding.

## Results and discussion

### Expression

The RBP gene was identified in the *T. maritima *genome sequence [17] as open reading frame (ORF) *tm0958*, based on sequence similarity to the *E. coli *RBP, and genetic linkage of this ORF within a putative operon that contains sequences for ABC transporters characteristic of a ribose transport system [18]. ORF *tm0958 *was amplified from *T. maritima *genomic DNA using the polymerase chain reaction. The resulting DNA fragment was cloned into a pET21a vector with a C-terminal hexa-histidine tag preceded by a glycine-serine linker. The nucleotide sequence of the recombinant was confirmed by DNA sequencing. Over-expression of this ORF in *E. coli *produced ~50 mg of pure protein per liter of growth medium, which was purified by immobilized metal affinity chromatography [19] followed by gel filtration chromatography.

**Figure 1:**
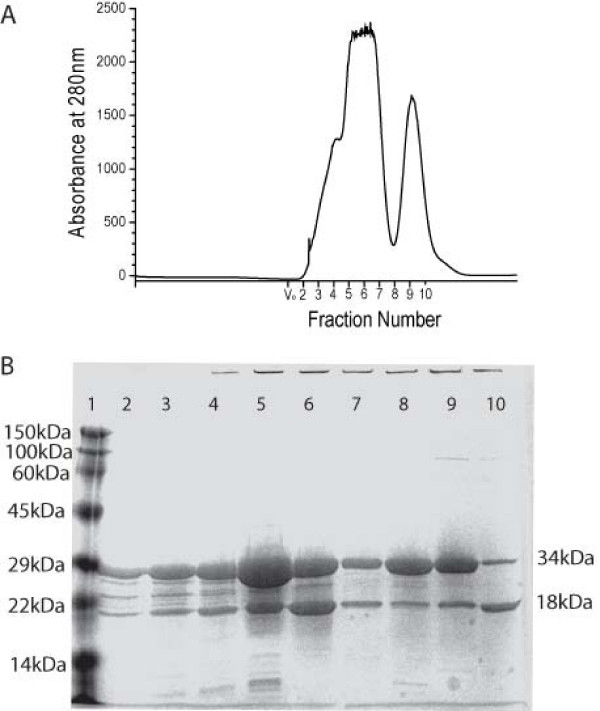
**Expression and purification of the tm0958 ORF**. (A) Gel-filtration (Superdex S75) chromatogram of the immobilized metal affinity purified tmRBP. Fractions (10 mL) and the void volume of the S75 column (V_o_) are indicated. (B) SDS-PAGE of column fractions. Lane 1 is a molecular mass ladder.

**Figure 2:**
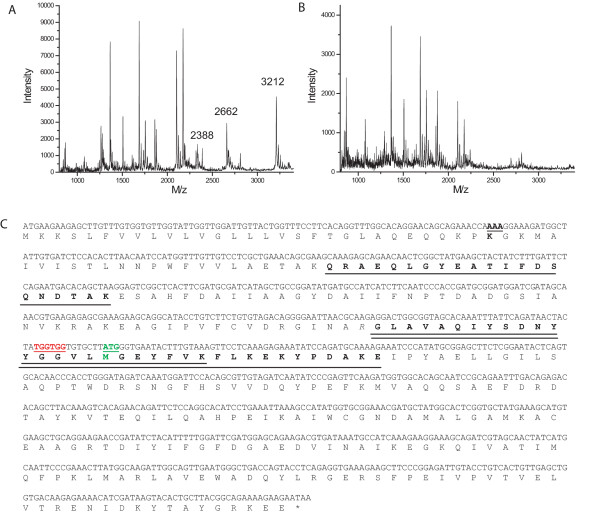
**Peptide mapping of the *tm0958 *ORF gene products**. MALDI mass spectra of the in-gel tryptic digests of the (A) 32 kDa and (B) 20 kDa products of the *tm0958 *ORF. Peptides observed in (A) that were not observed in (B) are indicated. (C) Mapping of the peptides from (A) onto the tmRBP amino acid and DNA sequence. Mapped peptides are underlined in black, met142 is underlined in green, and the alternate ribosome binding site is underlined in red.

The gel filtration elution profile of tmRBP consists of two peaks, one of which is consistent with a monomeric tmRBP (34 kDa), the other consistent with a ~55 kDa protein (Figure 1). SDS-PAGE of the resulting fractions revealed that both peaks contain tmRBP. The fractions corresponding to the 55 kDa protein also contain significant amount of a ~20 kDa species (Figure 1). Tryptic digestion of this 20 kDa protein, followed by MALDI mass spectrometry peptide mapping [20], revealed that it corresponds to a truncated form of the full-length tmRBP (Figure 2). The 55 kDa protein is therefore a heterodimer consisting of one full-length and one truncated copy of tmRBP. Neither full-length, nor truncated homodimers were observed. Analysis of the *tm0958 *DNA sequence suggests that this truncation may result from translation initiation at methionine 142 (numbering according to NCBI NP 228766), which is preceded by a ribosome binding site (Figure 2). This interpretation is further supported by the M142A mutant tmRBP in which the 20 kDa truncation is absent (data not shown).

### Thermal Stability

**Figure 3:**
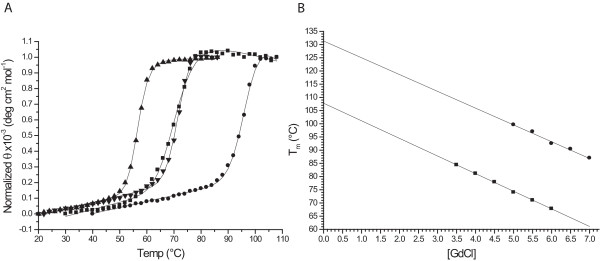
**Thermal stability of tmRBP**. (A) Thermal denaturation of tmRBP in 5.5 M GdCl (squares), tmRBP in 1 mM ribose and 5.5 M GdCl (circles), apo ecRBP (triangles), ecRBP in 1 mM ribose (inverted triangles). Solid lines in (A) are fit to a two-state model which takes into account the native and denatured baseline slopes [21,22]. (B) Extrapolated ^*app*^*T*_*m *_value of apo (squares) and ribose-bound (1 mM) (circles) tmRBP obtained from the series of thermal melting curves at different GdCl concentrations [23,26]. Solid line represents a linear fit to the observations.

The apparent thermal stability (^*app*^*T*_*m*_) of full-length monomeric wild-type tmRBP was determined by thermal denaturation using circular dichroism (CD) [21]. In the absence of denaturant, no significant change in the CD signal could be observed as a function of temperature (data not shown). All measurements were therefore carried out in the presence of the chemical denaturant guanidine hydrochloride (GdCl) to bring thermal denaturation into a measurable range. Melting curves were found to fit a two-state model [21,22]. An ^*app*^*T*_*m *_in the absence of GdCl was determined by linear extrapolation of a series of melting point determinations carried out at different GdCl concentrations [23] (Figure 3) and was found to be 108°C. tmRBP is significantly more stable than the mesophilic ecRBP (^*app*^*T*_*m *_value is 56°C (Figure 3)). Addition of the 20 kDa truncation has no effect on the ^*app*^*T*_*m *_value of the full-length wild-type monomeric protein (data not shown).

### Ligand Binding

Ribose binding was observed as a ligand-mediated change in the ^*app*^*T*_*m *_of full-length wild-type monomeric tmRBP in the presence of 5.5 M GdCl. Under these conditions the ^*app*^*T*_*m *_is 71°C in the absence of sugar and 97°C in the presence of 1 mM ribose, indicating that tmRBP is a ribose-binding protein, as predicted from sequence homology (Figure 3). For the ligand-bound form (1 mM ribose), an ^*app*^*T*_*m *_of 131°C in the absence of GdCl was determined by linear extrapolation of a series of melting point determinations carried out at different GdCl concentrations [23] (Figure 3).

### Structure Determination

Crystals of ribose-complexed tmRBP were grown using a full-length wild-type construct (residues 30–323) that lacks the periplasmic signal sequence (residues 1–29). The apo-protein was crystallized using a construct that consisted of residues 30–310 (numbering according to NCBI NP 228766), containing a M142A mutation to prevent expression of the in-frame ORF. We were unable to obtain crystals of the heterodimeric form. The apo-protein and ribose-complex diffract to 1.4 Å and 2.15 Å resolution and were refined to *R*_*cryst*_*/R*_*free *_values of 18.0/20.3 and 19.3/22.3 respectively. The X-ray crystal structure of ribose-bound tmRBP was solved by molecular replacement using ecRBP as the search model [24]. The apo-form of tmRBP was solved by separately searching with the amino- and carboxy-terminal domains of the ribose-bound form of tmRBP. Data collection, refinement, and stereochemistry statistics are summarized in Table 2.

**Table 2:** Data collection and refinement statistics.

	**tmRBP-apo**	**tmRBP-ribose**
**Data Collection**		
Wavelength (Å)	0.997	0.979
Resolution (Å)	1.40	2.15
Unique reflections	115460	25783
Mean I/σ(I)^a^	34.2 (1.7)	25.7 (3.6)
Completeness (%)^a^	99.0 (88.8)	80.9 (21.0)
R_sym _(%)^a^	5.0 (51.5)	5.6 (28.4)
Redundancy^a^	5.8 (3.4)	5.8 (1.6)
**Refinement**		
Resolution (Å)	50.0–1.40	50.0–2.15
Num. of Reflections (working set/test set)	115460/5767	23715/1354
R_cryst _(%)	18.0 (28.0)	19.3 (25.4)
R_free _^b ^(%)	20.3 (32.9)	22.3 (29.2)
Number of atoms		
Protein	4326	2286
Water	627	142
Ligand	0	10
**r.m.s.d**.		
Bond lengths (Å)	0.009	0.012
Bond angles (°)	1.2	1.2
**Average B-factor (Å^2^)**		
Main Chain	15.3	34.5
Side Chain	17.3	35.8
Solvent	29	37.7
Ligand		24.8
**Protein Geometry**		
Ramachandran outliers (%)	0.4	0.3
Ramachandran favored (%)	98.7	97.6
Rotamer outliers (%)	2.2	3.0

^a^Number in parentheses represent values in the highest resolution shell.

^b^R_free _is the R-factor based on 5% of the data excluded from refinement.

### Overall Structure and Comparison of the *E. coli *and *T. maritima *apo proteins

**Figure 4:**
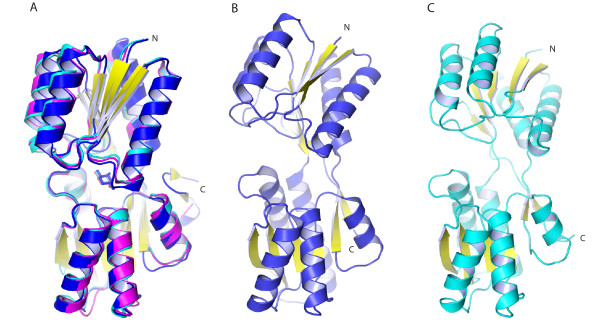
**Comparison of the *T. maritima *and *E. coli *RBP**. (A) Superimposition of ribose-complexed *T. maritima *(blue), *E. coli *(cyan) [24] and *T. tengcongensis *(magenta) [26] RBPs. (B) Ribbon representation of *T. maritima *RBP molecule A; (C) *E. coli *RBP [8]. N- and C-termini are indicated; yellow, β-strands; green, ribose. Structures in (B) and (C) are aligned on the C-terminal domain.

The apo forms of ecRBP [8] and tmRBP adopt the same overall fold. However, the relative inter-domain angles [25] differ significantly (43° for ecRBP; 28° and 20° for the two molecules in the tmRBP unit) (Figure 4). The hinge in ecRBP is very flexible as evidenced by the number of crystal forms that differ in the inter-domain closure angle [8]. The two molecules found in the tmRBP asymmetric unit differ in the inter-domain closure angle by 10°, analogous to the conformational heterogeneity observed in ecRBP [8].

The construct used to crystallize the apo-form of tmRBP was a C-terminally truncated form of the protein (13 amino acids). It is possible that the absence of this region could in some way influence the observed conformation of apo form of tmRBP. However, superimposition of the tmRBP ribose complex C-terminal domain onto the C-terminal region of the apo protein suggests that these this region does not form interdomain interactions in the absence of ligand.

### Overall Structure and Comparison of the *E. coli *and *T. maritima *ribose complexes

The structure of the tmRBP ribose-complex is similar to the ribose complexes observed in ecRBP [24] and a thermophilic RBP obtained from *Thermoanaerobacter tengcongensis *[26] (tteRBP). Both structures superimpose on tmRBP with a 1.2 Å RMSD calculated over C_α _atoms (Figure 4). The structures tteRBP and ecRBP are almost identical [26]; comparisons are described therefore only for ecRBP. The largest differences between ecRBP and tmRBP are at the C-termini, where tmRBP is extended by an additional 13 residues that are not present in ecRBP. This segment forms a short α-helix terminated by a β-hairpin (Figure 4). One of the amino acids in this region (Y289) forms extensive van der Waals interactions with the amino acids in the N-terminal domain (P14, W15 and V18). As similar extensions are found interacting with the N-terminal domain in both open and closed forms of other PBPs [27,28]. We postulate that these C-terminal extensions form inter-domain interactions that may be important for modulating the intrinsic free energy difference between the apo and closed forms in the absence of ligand (Miklos, Cuneo and Hellinga; in preparation).

**Figure 5:**
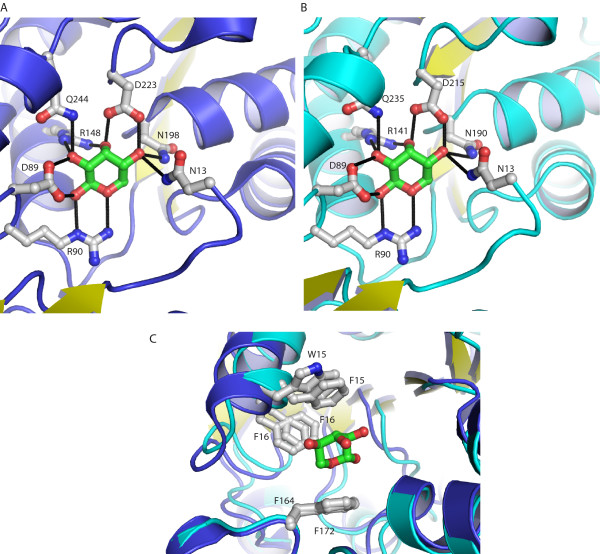
**Comparison of the ribose-complexes of *T. maritima *and *E. coli *RBPs**. Close-up view of polar amino acids (gray) in tmRBP (A) and ecRBP (PDB code 2DRI[24]) (B) that form a hydrogen-bonding network (black lines) with ribose (green). (C) Close-up view of the aromatic binding pocket residues of ecRBP (cyan) and tmRBP (blue). Phenylalanine (F15) in ecRBP is replaced by tryptophan (W15) in tmRBP. Superposition of the two structures reveals that the six-membered ring of the tmRBP tryptophan indole is coincident with the ecRBP phenylalanine six-membered ring.

Although ribose is commonly found as a furanose carbohydrate in biological molecules (e.g. nucleic acids), all periplasmic RBPs, including tmRBP, bind the β-anomer of D-pyranose ribose [24,26] (as initially postulated by Koshland [29]). β-D-pyranose ribose the most prevalent form in solution under ambient conditions (59%) [30]. The ligand-binding site of tmRBP is composed of a network of polar amino acids which is identical in sequence and hydrogen-bonding pattern to the *E. coli *protein [24] (Figure 5). Seven polar amino acids make a total of eleven hydrogen-bonds with the ribose. One residue in ecRBP (Q235) has been postulated to be important for both ligand-binding and hinge-bending; in the closed form it forms hydrogen-bonds with the ligand and amino acids from both domains [8,15]. The equivalent residue (Q244) and the amino acids which it interacts with are conserved in tmRBP. This pattern of conservation suggests that similar mechanisms couple ligand-binding to conformational changes in both proteins [8,14].

The ribose is wedged between three aromatic amino acids (W15, F16 and F172) which make extensive van der Waals interactions with the sugar ring. In ecRBP the equivalent aromatic binding pocket residues are all phenylalanines. Alignment of tmRBP and ecRBP structures indicates that the six-membered ring of W15 in tmRBP is equivalent to F15 in ecRBP (Figure 5).

### Open to Closed Transition: Global Changes

**Figure 6:**
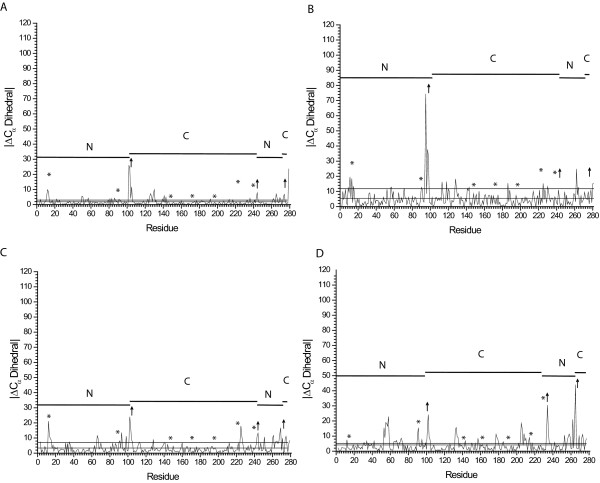
**Comparison of ligand-induced local conformational changes in the protein backbone of *T. maritima *and *E. coli *RBPs**. The absolute value of the change in the dihedral angle determined by four successive C_α _atoms is shown [41]. (A) Comparison between the A and B molecules of the *T. maritima *RBP apoprotein reveals that these two molecules differ primarily in the hinge region and represent different points along the hinge bending trajectory. (B) Comparison between molecule B of the apoprotein and the ribose-complexed tmRBP. (C) Comparison between molecule A of the apoprotein and the ribose-complexed tmRBP. (D) Comparison of the apo and ribose-complexed ecRBP (PDB code 1URP and 2DRI respectively). The span of the N- and C-terminal domains is indicated by solid horizontal lines; hinge regions are indicated by arrows. Regions near the binding pocket are marked by an asterisk. The mean of the |ΔCα| dihedral angle and one standard deviation away from the mean are indicated by a dashed and solid line respectively.

The addition of ribose to tmRBP induces a 28° hinge-bending motion [25] mediated about residues 102–105, 244–249, and 271–275. The hinge-bending motion of tmRBP is smaller than the 43° change observed in ecRBP [8]. In both ecRBP and tmRBP, the effects of these motions on the backbone are confined largely to the hinge region (Figure 6).

The two molecules in the tmRBP asymmetric unit have slightly different degrees of closure, indicative of an intrinsic flexibility of the hinge, as observed in ecRBP [8] and *E. coli *allose-binding protein [7]. Molecule B is related to molecule A by a 10° closing about the hinge. This movement is limited to one of the two strands (residues 101–106) which connect the two domains (Figure 6). The magnitude of C_α _torsion changes transitioning between the open and closed states is significantly greater for molecule B than molecule A (Figure 6); the average B-factors of the two molecules are the same.

### Open to Closed Transition: Local Changes

**Figure 7:**
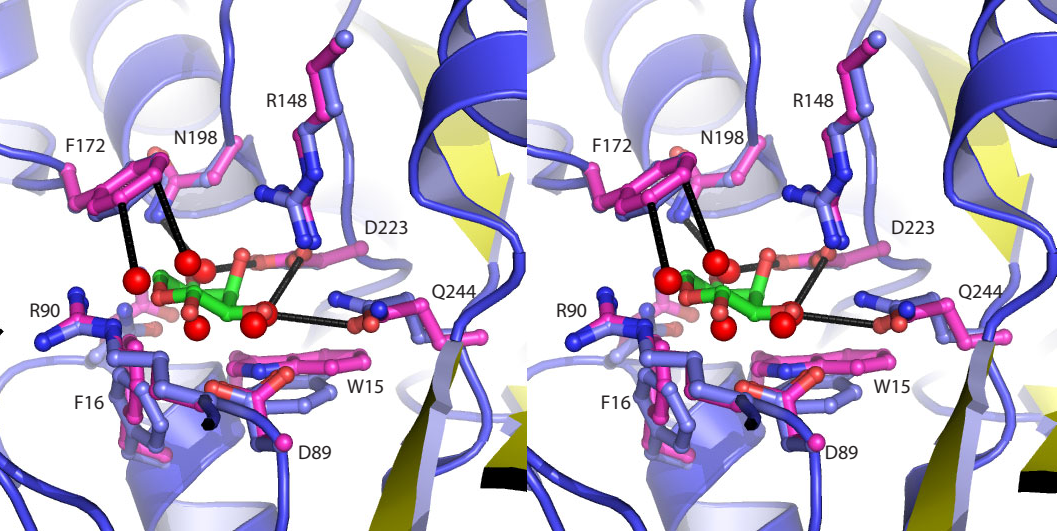
**Binding pocket organization of the apo and ribose-bound tmRBP**. Stereo-view of the ribose-bound tmRBP (blue) binding pocket superimposed with the binding pocket amino acids of apo tmRBP (magenta). The C-terminal residues of the apoprotein have similar rotamers as the ribose-bound form while the rotamers of the N-terminal domain apoprotein and ribose-bound forms are in different states. The C-terminal binding pocket residues of the apoprotein interact (black lines) with bulk solvent (red spheres) in a similar manner as the ligand-bound form does with the ribose ligand, pre-organizing the apo form.

In ecRBP and tmRBP local ligand-induced changes are restricted largely to the hinge region, the N-terminal amino acids that interact with ribose, and the hinge amino acid (Q235 and Q244 in ecRBP and tmRBP respectively) that interacts with the ribose (Figure 7 and Table 3). The amino acid side-chains in the C-terminal domain of tmRBP remain fixed in the same rotameric state in both apo and ligand-bound forms (Figure 7 and Table 3). By contrast, the side-chain torsional changes in the N-terminal domain of tmRBP are significant; in particular, W15 and F16 undergo torsional movements about χ_1 _and χ_2 _(Figure 7 and Table 3). This ligand-induced binding pocket rearrangement of the N-terminal domain is also observed in ecRBP, but of smaller magnitude than in tmRBP, and is restricted to three polar amino acids (N13, D89, and R90) (Table 3).

**Table 3:** Rotamers changes in the ecRBP (1URP molecule A/2DRI) and tmRBP (apoprotein molecule A/ribose-bound form) binding pocket residues.

	**ecRBP**					**tmRBP**				
	
	**Δχ1 (°)**	**Δχ2 (°)**	**Δχ3 (°)**	**Δχ4 (°)**	**(Σ|Δχ|)/N_χ_(°)**	**Δχ1 (°)**	**Δχ2 (°)**	**Δχ3 (°)**	**Δχ4 (°)**	**(Σ|Δχ|)/N_χ_(°)**
	
ASN13	12	-13			13	13	-1			7
PHE15	-1	16			8	26	-173			100
PHE16	-7	10			8	-17	26			22
ASP89	-18	17			18	-1	19			10
ARG90	26	-17	-27	70	35	-1	-5	5	0	3
**ARG141/148**	-1	-12	-7	-6	6	0	-12	4	-19	9
**PHE164/172**	0	0			0	1	4			3
**ASN190/198**	-7	-4			5	4	-8			6
**ASP215/223**	14	4			9	-1	6			3
↑GLN235/244	-20	-7	45		24	-3	9	-29		14

C-terminal amino acids are in bold face type; the hinge amino acid that interacts with ribose is indicated by an arrow. Where amino acid numbering differs, ecRBP residues are listed first.

### Solvent Interactions in tmRBP and ecRBP

Water molecules play an important role in the hinges of PBPs [7]. Analysis of the conservation pattern of bound water molecules among various PBPs identifies critical water molecules that participate in inter-strand hydrogen bonding in the hinge, in place of amino acid side-chains [7,8]. The positions of four bound water molecules are conserved (separated by less than 1.5Å in the aligned structures) in the open forms of ecRBP and tmRBP. One of these water molecules (HOH5 in tmRBP, W1 in ecRBP) is conserved in both the open and closed forms of group I PBPs [7]. This water molecule remains fixed in position in both the apo and ligand-bound forms. It is postulated to act as a "ball bearing" by serving as a fixed intra-hinge rotation point for the two domains [7]. It also mediates indirect interstrand hydrogen bonding. Another water molecule conserved among other group I PBPs, W2 [7], is absent from tmRBP. When present, this water mediates hydrogen-bonding between the first and last inter-domain strand in both the open and closed forms of group I PBPs. In tmRBP the conformation of the hinge strands permits direct inter-strand hydrogen-bonding between the main-chain nitrogen of residue 105 and the main-chain oxygen of residue 273, thereby replacing the contacts that would be made by W2 [7].

No water molecules interact directly with ribose in either ecRBP or tmRBP. Nevertheless, nine out of eleven water molecules within a 7Å sphere of the ribose are conserved among the sugar complexed forms of ecRBP and tmRBP. In the ecRBP ribose complex, W2 forms hydrogen-bonds with two of the hinge strands in the closed form. The opening motion of ecRBP forces out this solvent molecule, replacing the water-mediated hydrogen bonds with inter-strand hydrogen bonds [8]. W2 is absent from the ribose-bound tmRBP structure, as it is in *E. coli *arabinose-binding protein [7]. In both instances, the hinge conformation allows for inter-strand hydrogen-bonding to satisfy the water-mediated hydrogen-bonds that would be formed [7,8,24].

## Conclusion

We have characterized the ligand-binding properties of a putative ribose-binding protein identified in the genomic sequence of the extremophilic bacterium *T. maritima *and solved its X-ray crystal structure in the absence and presence of ribose. The structure reveals that tmRBP has high structural similarity to its mesophilic homolog ecRBP. Polar ligand interactions and ligand-induced global conformational changes are conserved [8,24]. Local structural rearrangements involving side-chain motions in the ligand-binding site differ in the mesophilic and thermophilic RBPs. In ecRBP the conformation of the binding pocket undergoes little ligand-induced rearrangement. The amino acids in the N-terminal domain of the tmRBP binding pocket undergo large χ_1 _and χ_2 _torsional changes, whereas the C-terminal domain remains fixed. Based on hydrogen-bonding pattern (6 and 5 hydrogen-bonds with the N- and C-terminal domains respectively) and buried surface area (55Å ^2 ^and 35Å ^2 ^with the N- and C-terminal domains respectively) it has been postulated that ordered binding occurs and ribose initially interacts with N-terminal domain of ecRBP [8]. If an order of interaction can be established from analysis of structure, it is likely to proceed with ribose initially interacting with the C-terminal domain of the apo tmRBP, as the entropic costs of fixing the side-chains for ligand binding should be reduced for a pre-ordered binding site.

Water molecules have been suggested to play an important mechanistic role in the evolution and adaptation of the PBP hinge [7]. In particular, two water molecules, (W1 and W2), are closely associated with the hinges of group I PBPs [7]. In tmRBP, W1, which is postulated to act as a "ball bearing" in the ligand-mediated conformational change, is conserved in both the apo- and ribose-bound forms. On the other hand, W2, which is involved in mediating important inter-hinge contacts in apo- and ligand-bound group I PBPs, is absent in both forms of tmRBP. In tmRBP the inter-strand hydrogen bonds form directly in the hinge. These differences in water interactions in the hinges of PBPs suggest local structural differences can supplant the need for W2, whereas the role of W1 cannot be accommodated through differences in main-chain geometry or side-chain identity.

Ligand-induced hinge bending motion is a key characteristic of the periplasmic binding protein superfamily. Analysis of PBP structures has provided a detailed description of this class of conformational change [7,12-14]. The detailed comparative analysis of the open to closed transition of the thermophilic tmRBP and mesophilic ecRBP presented here illustrates the subtle differences in the mechanism and magnitude of the ligand-induced conformational changes, and the interplay between global and local conformational changes in this protein superfamily.

## Methods

### Cloning Over-expression and Purification

The *tm0958 *gene was amplified from *T. maritima *genomic DNA (American Type Culture Collection) by the sticky-end PCR method [31] using the following primers to make the full-length tmRBP (residues 30–323) and the construct used to crystallize the apo form of tmRBP (residues 30–310) (numbering according to NCBI Protein Database NP 228766: PO^4-^-TATGAAAGGAA AGATGGCTATTGTGATCTCC and for the 5'-TGAAAGGAA AGATGGCTAT TGTGATCTCC end of the genes; PO^4-^-AATTCTA ATGGTGATGGTGATGGTGACTGCCTTCTTCTTTTCTGCCGTAAGCAGTG and CTAATGGTGATGGTGATGGTGACTGCCTTCTTCTTTTCTGCCGTAAGCAGTG for the 3'end of the full-length tmRBP gene, PO^4^-AATTCTAATGGTGATGGTGATGGTGACTGCCTTCTCTTGTCACCAGCTCAACAGTGAC and CTAATGGTGATGGTGATGGTGACTGCCTTCTCTTGTCACCAGCTCAACAGTGA C for the 3' end of the tmRBP-apo gene [31]. The 30–323 construct which was used to crystallize the apo-form additionally contains an M142A mutation to prevent translation of the truncated form of tmRBP. The resulting fragments were cloned into the NdeI/EcoRI sites of a pET21a (Novagen) plasmid for over-expression in *E. coli*. This ORF lacks the periplasmic signal sequence. The coding sequence starting at lysine 30 was cloned in-frame with an ATG start codon. A hexa-histidine affinity tag, preceded by a glycine-serine linker, was fused in-frame at the carboxy terminus to facilitate purification by immobilized metal affinity chromatography (IMAC). Protein concentration was determined spectrophotometrically (ε _280 _= 41,000 M^-1^cm^-1^) [32]. The resulting gene product was expressed and purified by IMAC and gel filtration as described [23]. Pooled IMAC fractions were concentrated to 12 ml and were loaded onto a Superdex 26/60 S75 (Amersham) gel filtration column that was previously that was previously calibrated with blue dextran, bovine serum albumin, chicken serum albumin, chymotrypsin and lysozyme.

### Tryptic Digest and Mass Spectrometry

Proteins were excised from a 12% Tris-HCl SDS-PAGE gel and were digested in-gel using the Pierce In-gel Tryptic Digest Kit. Mass spectra were acquired on an Applied Biosystems Voyager DE MALDI-TOF mass spectrometer using an α-cyano-4-hydroxycinnamic acid matrix with a 300 ns delay time.

### Circular Dichroism

Circular dichroism (CD) measurements were carried out on an Aviv Model 202 CD spectrophotometer. Thermal denaturations were determined by measuring the CD signal at 222 nm (1 cm path length) as a function of temperature, using 1.0 μM of full-length wild-type monomeric tmRBP (10 mM Tris-HCl pH 7.8, 150 mM NaCl) in the presence or absence of 1 mM ribose at several GdCl concentrations extrapolated to 0 M GdCl [23]. Protein samples were incubated for 15 minutes prior to collecting data. Each measurement includes a 3-second averaging time for data collection and a 60 second equilibration period at each temperature. Data were fit to a two-state model [22].

### Crystallization and Data Collection

Crystals of full-length wild type ribose-complexed tmRBP were grown using 3:1 stoichiometric ribose:protein ratio by micro-batch under paraffin oil in drops that contained 2 μl of the protein solution (15 mg/ml in 10 mM Tris pH 7.8, 20 mM NaCl, 1.5 mM ribose) mixed with 2 μl of 0.1 M MES pH 6.0, 20% (w/v) PEG 8000 and 0.1 M RbCl. Crystals of the C-terminally truncated M142A apoprotein were grown in micro-batch drops containing 2 μl of the protein solution (15 mg/ml in 10 mM Tris pH 7.8, 20 mM NaCl) mixed with 2 μl of 0.1 M Bis-Tris pH 5.9, 25% (w/v) PEG 3350, 0.2 M NaCl. Diffraction quality crystals typically grew within two weeks at 17.0°C. The ribose-complexed crystals diffract to 2.15 Å resolution and belong to the I222 space group (*a *= 72.1 Å, *b *= 98.2 Å, *c *= 131.1 Å) (Table 2). The apo tmRBP crystals diffract to 1.4 Å resolution and belong to the F222 space group (*a *= 120.9 Å, *b *= 136.8 Å, *c *= 144.5 Å) (Table 2). Crystals were transferred stepwise to a cryoprotectant solution consisting of the original precipitant solution with an additional 15% ethylene glycol or glycerol, after which they were mounted in a nylon loop and flash cooled in liquid nitrogen. All data were collected at 100 K on the SER-CAT 22ID beam line at the Advanced Photon Source. Diffraction data were scaled and integrated using HKL2000 [33].

### Structure Determination Methods, Model Building and Refinement

The structure of ribose-complexed tmRBP was determined by molecular replacement utilizing the AMore program, where the ligand-bound form of the *E. coli *ribose-binding protein was used as the search model [34]. The N- and C-terminal domains of ribose-complexed tmRBP were used as a search model in Phaser to solve the apoprotein structure [35]. In both cases, rotation, translation, and fitting functions revealed a single clear solution yielding higher correlation coefficients and a lower *R *factor than all the others. Manual model building was carried out in the programs O and COOT and refined using REFMAC5 [36-38].

### Structural Analysis

The final model for ribose-complexed tmRBP includes one intact monomer (residues 30–323), one ribose molecule, and 142 water molecules. The final model for the apoprotein includes two intact monomers (residues 30–310) and 627 water molecules. The models exhibit good stereochemistry as determined by PROCHECK [39] and MolProbity [40]; final refinement statistics are listed in Table 2. PDB coordinates and structure factors of ribose-complexed tmRBP and apoprotein have been deposited in the RCSB Protein Data Bank under the accession codes 2FN8 and 2FN9 respectively.

Large-scale hinge bending motions were analyzed with the DynDom web server [25]. Local C-alpha torsional changes were analyzed with LSQMAN [41].

## Authors' contributions

MJC purified, crystallized, solved the structure of tmRBP, and carried out circular dichroism experiments. MJC, LSB and HWH undertook sequence and structural analysis of the tmRBP and ecRBP structures. MJC and HWH wrote the manuscript. All authors have read and approved the final manuscript.
